# The torsion of a wandering pelvic spleen: A case report

**DOI:** 10.1186/1757-1626-1-149

**Published:** 2008-09-10

**Authors:** Francesco Feroci, Egidio Miranda, Luca Moraldi, Renato Moretti

**Affiliations:** 1 Division of General Surgery, Ospedale Misericordia e Dolce, P.za dell'ospedale 5, 59100 Prato, Italy; 2 Division of General Surgery, AOU Careggi, V.le Morgagni 85, 50100 Firenze, Italy

## Abstract

A 15 years old patient was taken to the operative room for an explorative laparotomy due to abdominal pain and a pelvic spleen at preoperative computed tomography: was pointed out the absence of all splenic ligamentous attachments and short gastric vessels with a consequently dislocation of a bigger and congested spleen in the pelvis. This organ, wrapped in the omentum, was in a serious ischemic suffering due to a 720 degrees clock torsion around its exceptionally long pedicle (about 20 cm); besides was confirmed pancreatic body and tail ectopy. Following the derotation, the volume of the organ has decreased but became fixed in above norm dimensions. A total splenectomy was executed.

## Case presentation

A 15 year old otherwise healthy Caucasian male student (52 kilograms of weight, 170 centimeters of height, no smoker, no drinking alcohol, not assuming medications with no significative family history) presented to the Emergency Department with a 24 hours abdominal pain and increase of his abdominal girth. The pain was non-continuous, poorly localized, and was non-colicky and non-radiating in nature. There was no history of vomiting, bowel or urinary symptoms. Over the prior 2 months, he reported one other similar episode that had resolved spontaneously. He was afebrile and his vital signs were stable. Abdominal examination revealed marked diffuse abdominal tenderness and guarding. A large hypogastric abdominal mass was palpable. Urinalysis and urine culture were normal; he had a white cell count of 19.4 × 10^9^/L, hemoglobin 11.0 g/dL and platelets 410 × 10^9^/L). On abdominal sonography, no spleen could be demonstrated in the normal position; the left upper quadrant was filled with bowel loops. An enlarged spleen extending from lower-polar region of the left kidney to the pelvis was seen. A large hypoechoic area was seen, suggestive of infarction, with a streak of perisplenic fluid. Ultrasonography demonstrated no blood flow in the splenic vein and in the splenic artery. Subsequent contrast-enhanced CT scan also showed absence of the spleen in the left upper quadrant as well as an enlarged spleen in the left lumbar and iliac region. The splenic parenchyma showed poorly, inhomogenous enhancing areas suggestive of infarction. The splenic vein was dilatated and showed a non-enhancing filling defect near the hilum, indicating the presence of a thrombosis. The splenic vessels, pancreatic tail, and the surrounding fat formed a whorled appearance, supermedial to the splenic hilum, that was suggestive of torsion.

**Figure 1:**
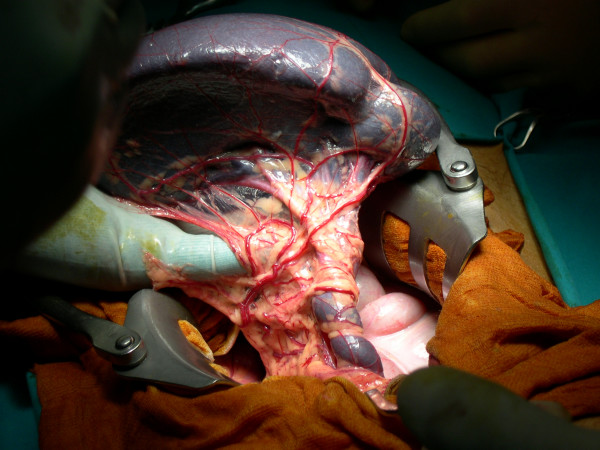
The spleen at the opening of the abdomen.

**Figure 2:**
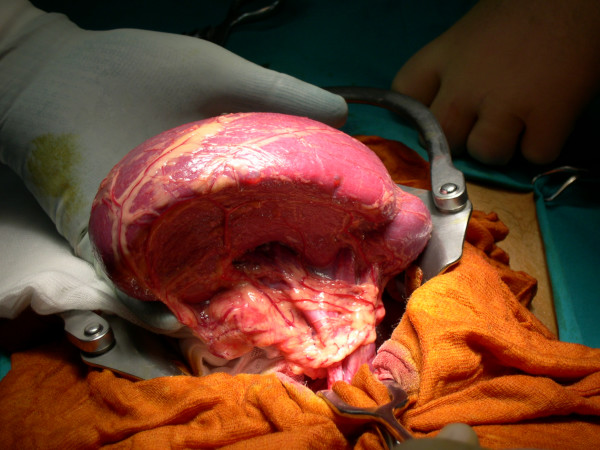
The spleen after the derotation.

The patient underwent exploratory laparotomy through a midline incision. This revealed the absence of all splenic ligamentous attachments and short gastric vessels with a consequently dislocation of a bigger and congested spleen in the pelvis. This organ, wrapped in the omentum, was in a serious ischemic suffering due to a 720° clock torsion around its exceptionally long pedicle (≈ 20 cm); besides was confirmed pancreatic body and tail ectopy (Figure 1). Following the derotation, the volume of the organ has decreased but became fixed in above norm dimensions (Figure 2). A total splenectomy was performed in view of a symptomatic wandering spleen, with hypersplenism and portal hypertension. The patient's post-operative course was uneventful.

## Discussion

A wandering spleen is a rare but well-known entity. The incidence is < 0.2%. It is more common in females than males in an adult population [1]. Acute, chronic or intermittent torsion of the spleen is the major complication of an abnormally mobile spleen, the "wandering spleen." The increased mobility of the spleen results from absence or laxity of the supporting ligaments (gastrosplenic and splenorenal ligaments) that normally anchor the spleen in its normal position [2].

The clinical presentation of a wandering spleen can be variable. Affected patients may be asymptomatic and this condition may be discovered incidentally as an abdominal mass on physical examination or on imaging for other unrelated reasons. Patients may have mild intermittent abdominal pain due to splenic congestion with intermittent torsion and spontaneous detorsion, or may present with an acute abdomen due to torsion of the splenic pedicle with subsequent infarction. With acute torsion, the condition can be confused with appendicitis or ovarian torsion. Other clinical symptoms include nausea, vomiting, fever, leukocytosis, peritoneal signs, and a palpable mass in the abdomen or pelvis [3].

For the definitive diagnosis of a wandering spleen, various imaging techniques, including plain radiography, barium enema, scintigraphy, gray-scale sonography, Doppler ultrasonography, CT, and angiography have been used [4].

Historically, splenectomy has been the treatment for symptomatic wandering spleen. With increasing appreciation for the importance of the spleen in reticuloendothelial function, there has been renewed interest in splenopexy. However, in cases of splenic torsion with infarction, splenectomy is required. Attention to vaccination for encapsulated organisms should be performed, usually 1 to 2 weeks after splenectomy [5].

## Consent

Written informed consent was obtained from the patient's mother for publication of this case report and accompanying images. A copy of the written consent is available for review by the Editor-in-Chief of this journal.

## Competing interests

The authors declare that they have no competing interests.

## Authors' contributions

EM recorded the patient data and took the photos. RM and LM performed the intervention, and FF was the major contributor in writing the manuscript. All authors read and approved the final manuscript.
